# Phase Morphology, Mechanical, and Thermal Properties of Calcium Carbonate-Reinforced Poly(L-lactide)-*b*-poly(ethylene glycol)-*b*-poly(L-lactide) Bioplastics

**DOI:** 10.3390/polym15020301

**Published:** 2023-01-06

**Authors:** Prasong Srihanam, Wiriya Thongsomboon, Yodthong Baimark

**Affiliations:** Biodegradable Polymers Research Unit, Department of Chemistry and Centre of Excellence for Innovation in Chemistry, Faculty of Science, Mahasarakham University, Mahasarakham 44150, Thailand

**Keywords:** poly(lactic acid), poly(ethylene glycol), block copolymer, calcium carbonate, reinforcing filler

## Abstract

Poly(L-lactide) (PLLA) is a promising candidate as a bioplastic because of its non-toxicity and biodegradability. However, the low flexibility of PLLA limits its use in many applications. Poly(L-lactide)-*b*-poly(ethylene glycol)-*b*-poly(L-lactide) (PLLA-*b*-PEG-*b*-PLLA) block copolymer is of interest for bioplastic applications due to its superior flexibility compared to PLLA. The aim of this work is to modify PLLA-*b*-PEG-*b*-PLLA using a low-cost calcium carbonate (CaCO_3_) filler to improve material properties compared to PLLA/CaCO_3_ composites. The addition of CaCO_3_ enhanced the crystallinity and thermal stability for the PLLA-*b*-PEG-*b*-PLLA matrix but not for the PLLA matrix, as determined by differential scanning calorimetry (DSC), X-ray diffractometry (XRD), and thermogravimetric analysis (TGA). Phase morphology investigation using scanning electron microscopy (SEM) revealed that the interfacial adhesion between PLLA-*b*-PEG-*b*-PLLA and CaCO_3_ was stronger than between PLLA and CaCO_3_. Additionally, tensile testing was carried out to determine the mechanical properties of the composites. With the addition of CaCO_3_, the tensile stress and Young’s modulus of the PLLA-*b*-PEG-*b*-PLLA matrix were increased, whereas these properties of the PLLA matrix were significantly decreased. Thus, CaCO_3_ shows great promise as an inexpensive filler that can induce nucleation and reinforcing effects for PLLA-*b*-PEG-*b*-PLLA bioplastics.

## 1. Introduction

Nowadays, bio-based polymers are gaining great interest for production of environmentally sustainable products due to their lower carbon footprint compared with petro-based polymers [1,2]. Among bio-based polymers, poly(L-lactic acid) or poly(L-lactide) (PLLA) has received the most attention because of its biodegradability, biocompatibility, processability, and possibilities for production scale-up [3,4,5]. PLLA has been extensively investigated for medical, pharmaceutical, packaging, agricultural, automotive, and electronic applications [1,2,5,6,7]. The main limitation in the use of PLLA is that it is less flexible [8]. High-molecular-weight PLLA-*b*-poly(ethylene glycol)-*b*-PLLA (PLLA-*b*-PEG-*b*-PLLA) block copolymers are more flexible than PLLA due to flexibility of the PEG middle-blocks [9,10].

PLLA composites have been prepared in combination with micro- and nano-fillers to improve some properties and/or to reduce the production cost of the products [1,11,12,13]. Many mineral fillers such as talc, calcium carbonate, kaolin, and silicon dioxide have been used for this purpose [1,14]. Calcium carbonate (CaCO_3_) is the cheapest mineral filler and is commonly available in the market. Unfortunately, weak interfacial adhesion with poor phase compatibility between PLLA matrix and CaCO_3_ filler was shown to be the main limitation for improving the properties of PLLA composites [15,16]. Modification of CaCO_3_ has been performed to make a hydrophobic surface to improve phase compatibility with the PLLA matrix [1,15,16,17,18,19]. The addition of surface-modified CaCO_3_ enhanced the thermal stability and the mechanical properties of the PLLA composites [1,15]. Therefore, improving phase compatibility between PLLA and CaCO_3_ remains a challenging area of research.

The hydrophilicity of the PLLA-*b*-PEG-*b*-PLLA was higher than that of the PLLA due to the hydrophilic PEG blocks [20]. The phase compatibility between the PLLA-*b*-PEG-*b*-PLLA matrix and hydrophilic thermoplastic starch (TPS) [20] was found to be better than that between the PLLA matrix and hydrophilic TPS. However, the combination of the PLLA-*b*-PEG-*b*-PLLA block copolymer with CaCO_3_ has not been reported so far. It could be conjectured that the hydrophilic PEG blocks could enhance the interfacial adhesion between the block copolymer and CaCO_3_. Thus, the aim of this research is to investigate the effect of the addition of a CaCO_3_ and CaCO_3_ ratio on thermal transition, thermal decomposition, and mechanical properties of the block copolymer-based composites. The composites were prepared by melt compounding block copolymer with CaCO_3_. The PLLA/CaCO_3_ composites were also prepared under the same condition for comparison.

## 2. Materials and Methods

### 2.1. Materials

PLLA-*b*-PEG-*b*-PLLA block copolymer was synthesized by ring-opening polymerization of L-lactide (LLA) monomer in the presence of a chain extender (2.0 parts per hundred of resin, phr) as described in our previous work [21]. The block copolymer was synthesized at 165 °C for 6 h under a nitrogen atmosphere using a stannous octoate/PEG mixture as the initiating system. The molecular weight of PEG was 20,000. Joncryl ADR 4368 (BASF, Bangkok, Thailand) was used as a chain extender. The number-averaged molecular weight (*M_n_*) and dispersity (*Đ*) of the block copolymer were characterized using a gel permeation chromatography (GPC, Waters e2695 separations module, Waters Corporation, Midford, MA, USA) equipped with PLgel 10 μm mixed B 2 columns operating at 40 °C at a flow rate of 1.0 mL/min. A refractive index (RI) detector was employed. Tetrahydrofuran was used as the solvent. The GPC curve of the block copolymer is presented in Figure S1. The results of *M_n_* and *Đ* were 108,500 and 2.2, respectively. The chemical composition of the block copolymer was determined as an LLA:ethylene oxide (LLA:EO) ratio using a ^1^H-NMR spectrometer (DPX 400, Bruker Advance, Karlsruhe, Germany) at 25 °C, as shown in Figure S2, from methine protons of LLA units (peak 1) and methylene protons of EO units (peak 14), repeating units of PLLA and PEG blocks, respectively [10]. CDCl_3_ was used as the solvent, and tetramethysilane was used as the internal standard. The resulting LLA:EO ratio was 60:40 mol%. Peaks 6–9 and 11–13 of a chain extender were also assigned [10].

**Figure 1:**
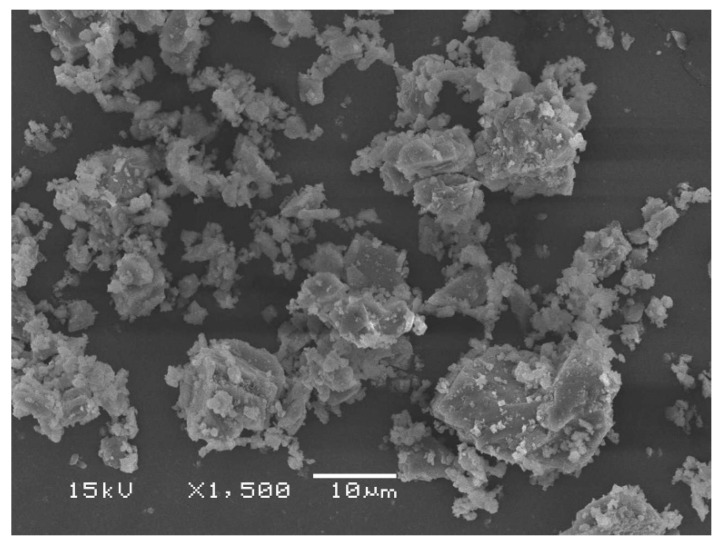
SEM image of CaCO_3_ powder.

PLLA (3251D grade) was purchased from NatureWorks LLC (MA, USA). Its *M_n_* and *Đ* were 56,200 and 1.6, respectively [22]. Melt flow indices (*MFI*) of PLLA and block copolymer determined at 190 °C under 2.16 kg load were 29 and 26 g/10 min, respectively. Calcium carbonate (CaCO_3_, 1250 grade) sieved with 1250 mesh was purchased from Thai Poly Chemicals Co., Ltd. (Samut Sakorn, Thailand). The SEM image of CaCO_3_ is shown in Figure 1.

### 2.2. Preparation of the Composites

Block copolymer and CaCO_3_ were dried in a vacuum oven at 50 °C for 24 h to remove moisture prior to melt compounding using a torque rheometer (HAAKE^TM^ Polylab OS Rheomix Thermo Scientific, Waltham, MA, USA) at 170 °C for 8 min with a rotor speed of 100 rpm. Block copolymer-based composites were prepared with 0%, 5%, 10%, 20%, and 30 %wt CaCO_3_. PLLA/CaCO_3_ composites were also prepared by the same conditions for comparison. All the obtained composites were dried in a vacuum oven at 50 °C for 24 h to remove moisture prior to processing into film (10 mm × 10 mm × 0.2 mm) using a hot-press machine (Carver Auto CH, Wabash, IN, USA). Each composite film was prepared by preheating at 170 °C for 4 min before hot pressing for 2 min under 5 MPa force. The resulting film was cooled with water-cooled plates for 1 min under 5 MPa force and was subsequently stored in a desiccator for at least 24 h before characterization.

### 2.3. Characterization of the Composites

The FTIR spectra of the samples were recorded using a FT-IR spectrophotometer (Invenio-S, Bruker, Karlsruhe, Germany) with the ATR accessory. A scan range of 4000 cm^−1^ to 500 cm^−1^ with a resolution of 4 cm^−1^ for 64 scans was used.

Thermal transition properties of the samples were studied using a differential scanning calorimeter (DSC, Pyris Diamond, PerkinElmer, Waltham, MA, USA). For DSC heating scans, the sample was first heated at 200 °C for 3 min to remove thermal history before fast quenching to 0 °C. Subsequently, the sample was scanned from 0 °C to 200 °C with a heating rate of 10 °C/min under a nitrogen gas flow. The degree of crystallinity from DSC (*DSC* − *X_c_*) of the sample was calculated from the following equation.

*DSC* − *X_c_* (%) = [(Δ*H_m_* − Δ*H_cc_*)/(93.6 × *W_PL__LA_*)] × 100 (1)

where ∆*H_m_* and ∆*H_cc_* are melting and cold-crystallization enthalpies, respectively. The Δ*H_m_* value for 100%*DSC* − *X_c_* PLLA is 93.6 J/g [20,23]. *W_PL__LA_* is the PLLA weight-fraction.

For DSC cooling scans, the thermal history of the sample was first removed by complete melting at 200 °C for 3 min. After that, the sample was scanned from 200 °C to 0 °C at a cooling rate of 10 °C/min under a nitrogen gas flow.

The thermal decompositions of the samples were determined using a thermogravimetric analyzer (TGA, SDT Q600, TA Instruments, New Castle, DE, USA). The sample was heated from 50 °C to 600 °C at a rate of 20 °C/min under a nitrogen gas flow.

The crystalline structures of the film samples were investigated using a wide-angle X-ray diffractometer (XRD, D8 Advance, Bruker Corporation, Karlsruhe, Germany). A CuKα radiation at 40 kV and 40 mA and a scan speed was 3 °/min were employed. The degree of crystallinity from XRD (*XRD* − *X_c_*) for PLLA crystallites was calculated with the following equation.

*XRD* − *X_c_* (%) = [(*A_c_*)/(*A_c_* + *A_a_*)] × 100 (2)

where *A_c_* and *A_a_* are the integrated peak areas for PLLA crystallites and the integrated halo area for the PLLA amorphous, respectively.

Phase morphology of the film cross-sections was investigated using a scanning electron microscope (SEM, JSM-6460LV, JEOL, Tokyo, Japan). The composite films were cryogenically fractured after immersing in liquid nitrogen. High-quality SEM images were obtained by sputter gold coating before SEM analysis at 15 kV.

The tensile properties of the film samples (80 mm × 10 mm) were measured using a tensile tester (LY-1066B, Dongguan Liyi Environmental Technology Co., Ltd., Guangdong, China) with a load cell of 100 kg according to the ASTM D638. The gauge length was 50 mm and tensile rate was 50 mm/min. The averaged tensile value was calculated from at least 10 specimens of each sample.

## 3. Results and Discussion

### 3.1. FTIR

**Figure 2:**
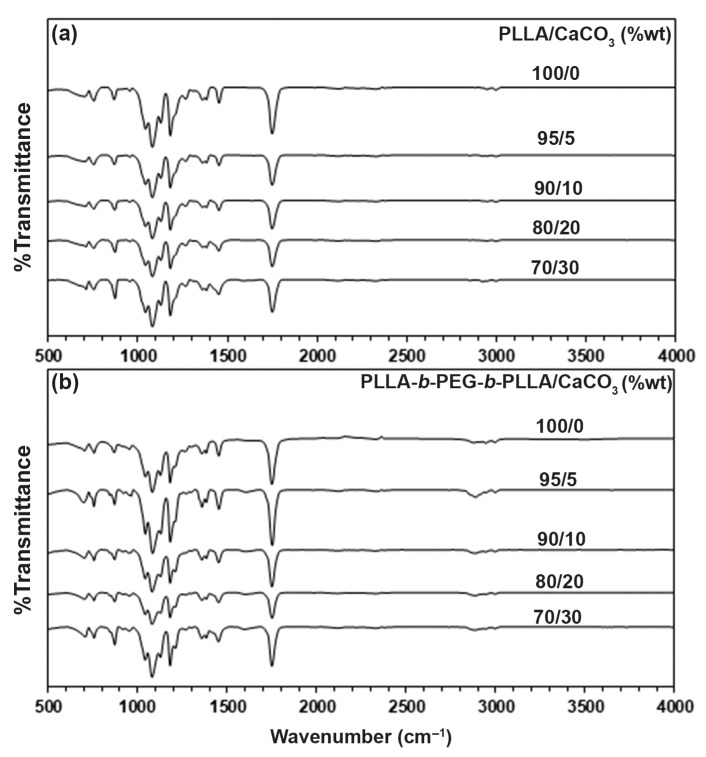
ATR-FTIR spectra of (**a**) PLLA/CaCO_3_ and (**b**) PLLA-*b*-PEG-*b*-PLLA/CaCO_3_ composite films with various CaCO_3_ ratios.

The chemical functional groups of the composites were determined from the ATR-FTIR spectra, as shown in Figure 2. The pure PLLA in Figure 2a exhibited a peak at 1748 cm^−1^, which was attributed to the carbonyl (C=O) in ester groups. The peaks at 1181 cm^−1^, 1128 cm^−1^, and 1080 cm^−1^ were assigned to C-O-C in ester groups and three peaks at 2995 cm^−1^, 2944 cm^−1^, and 2879 cm^−1^ were attributed to C-H stretching groups, which indicates the PLLA functional groups [20,24]. The pure block copolymer in Figure 2b showed peaks that were similar the pure PLLA with the addition of a peak at 2877 cm^−1^, which was attributed to methylene (−CH_2_) in oxyethylene groups (repeating units of PEG blocks) and a broad peak at 3501 cm^−1^, which was assigned to hydroxyl (−OH) end-groups [20].

The ATR-FTIR spectrum of CaCO_3_ is presented in Figure S3. There were three characteristic peaks of CaCO_3_ at 1398 cm^−1^, 873 cm^−1^, and 712 cm^−1^, which were attributed to the asymmetric stretching, in-plane bending, and out-of-plane bending modes of carbonate ions, respectively [17]. However, these peaks of CaCO_3_ were not clearly detected for the composites. This may be because they overlapped with the peaks from the PLLA and block copolymer matrices. From Figure S4, the −CH_2_ peak of block copolymer matrix at 2877 cm^−1^ shifted significantly to a higher wavenumber at 2886 cm^−1^ when the 5 %wt CaCO_3_ was initially loaded. However, this peak shifted to a lower wavenumber (2880–2881 cm^−1^) when the CaCO_3_ ratio was higher than the 5 %wt. On the other hand, the ATR-FTIR spectra of the PLLA-based composites did not change significantly with different CaCO_3_ ratios. This suggests the occurrence of interactions between the block copolymer and CaCO_3_ components. This assumption was clarified by SEM analysis.

### 3.2. Thermal Transition Properties

**Figure 3:**
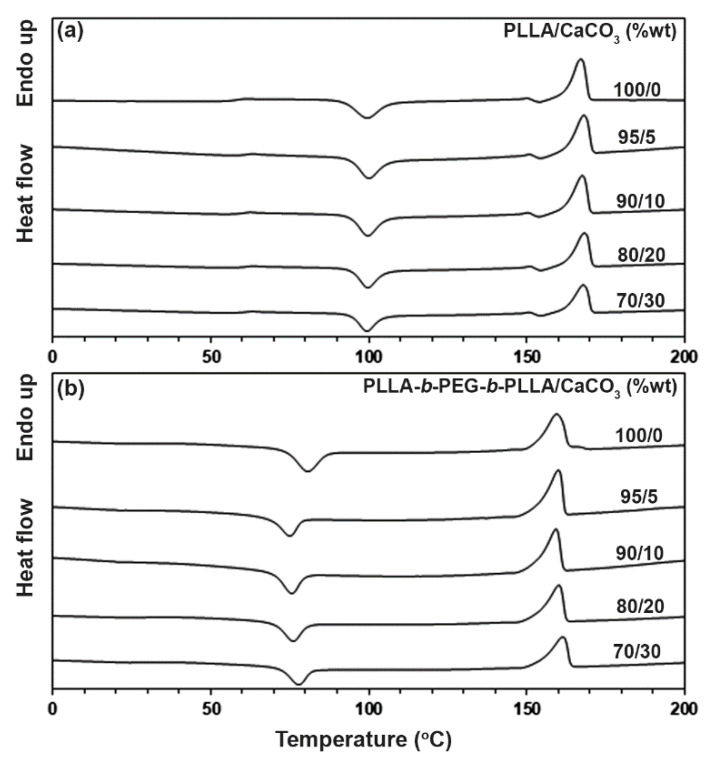
DSC heating curves of (**a**) PLLA/CaCO_3_ and (**b**) PLLA-*b*-PEG-*b*-PLLA/CaCO_3_ composites with various CaCO_3_ ratios.

The thermal transition properties of the composites were determined by the DSC method. Figure 3 shows the DSC heating curves of the composites with and without CaCO_3_, and Table 1 sums up the DSC results from Figure 3. The *T_g_* values of the PLLA-based and block copolymer-based composites were in the ranges of 58–60 °C and 30–32 °C, respectively. The *T_cc_*, *T_m_*, and *DSC* − *X_c_* values of the PLLA-based composites did not change significantly as the CaCO_3_ ratio increased. For the block copolymer-based composites, it should be noted that the *T_m_* peak of PEG with a molecular weight of 20,000 (about 68 °C) in the block copolymer was not detected [25]. This may be because the good miscibility between the PEG middle-blocks and the long PLLA end-blocks prevented the crystallization of PEG blocks [9,10]. When the 5 %wt CaCO_3_ was loaded, the *T_cc_* peak of the block copolymer dramatically decreased from 81 °C to 75 °C and the *DSC* − *X_c_* value increased from 13.9% to 18.4%. The shifting to lower temperature of the *T_cc_* peak suggests CaCO_3_ acted as a heterogeneous nucleating agent for the block copolymer [26]. However, when the CaCO_3_ ratio was higher than 5 %wt, the *T_cc_* peak of the block copolymer-based composites slightly shifted to a higher temperature and the *DSC* − *X_c_* value slightly decreased. This may have been due to the reduced nucleation efficiency from the aggregation at high ratios of the CaCO_3_.

**Figure 4:**
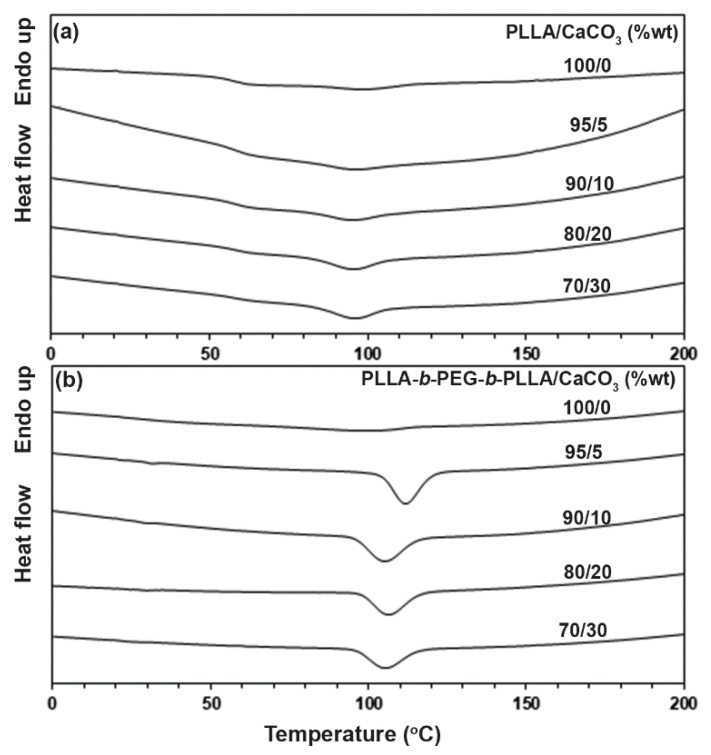
DSC cooling curves of (**a**) PLLA/CaCO_3_ and (**b**) PLLA-*b*-PEG-*b*-PLLA/CaCO_3_ composites with various CaCO_3_ ratios.

Figure 4 shows the DSC cooling curves of the composites. The DSC results from Figure 4 are summarized in Table 2. The *T_c_* peaks (95–99 °C) and Δ*H_c_* values (5.5–6.1 J/g) of the PLLA-based composites did not change significantly as the CaCO_3_ ratio increased. The *T_c_* peak of the block copolymer dramatically shifted from 99 °C to 112 °C and the Δ*H_c_* value greatly increased from 11.7% to 31.7% when the 5 %wt CaCO_3_ was loaded, indicating the nucleation efficiency of CaCO_3_ [23]. However, the *T_c_* peak of the block copolymer-based composites shifted to a lower temperature and the Δ*H_c_* value decreased when the CaCO_3_ ratio was higher than the 5 %wt. The results confirmed that CaCO_3_ acted as a heterogeneous nucleating agent enhancing the crystallization of the block copolymer, consistent with the previous discussed results from the DSC heating curves.

### 3.3. Thermal Decompositions

**Figure 5:**
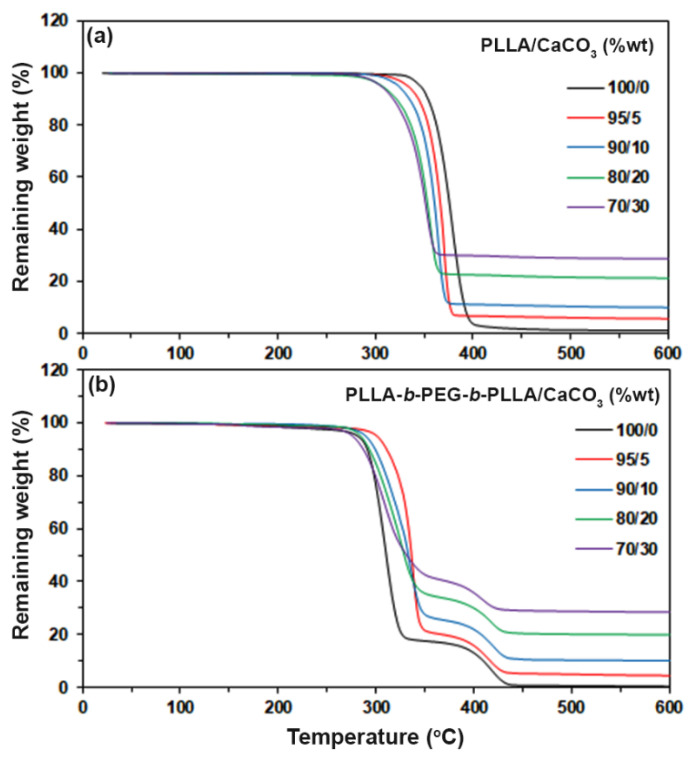
TG thermograms of (**a**) PLLA/CaCO_3_ and (**b**) PLLA-*b*-PEG-*b*-PLLA/CaCO_3_ composites with various CaCO_3_ ratios.

**Figure 6:**
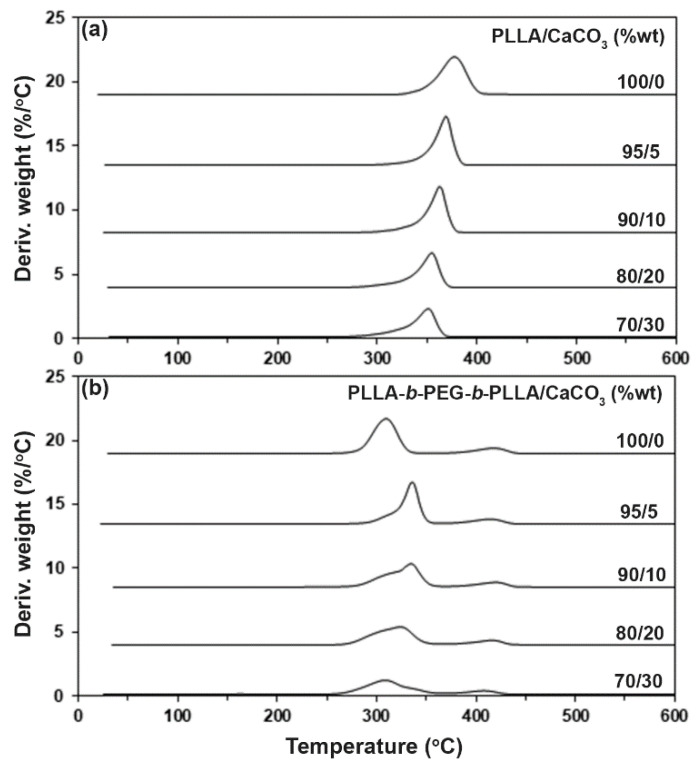
DTG thermograms of (**a**) PLLA/CaCO_3_ and (**b**) PLLA-*b*-PEG-*b*-PLLA/CaCO_3_ composites with various CaCO_3_ ratios.

The thermal decomposition behaviors of the composites were determined by the TGA method. Figure 5 shows the TG thermograms of the composites, and Table 3 sums up the *5%–T_d_* and residue ash at 600 °C values of the composites. Derivative TG (DTG) thermograms in Figure 6 were derived from Figure 5 to provide further details on the thermal decomposition behaviors. Each DTG peak was assigned to the *T_d,max_* peak of each thermal decomposition step, which is also reported in Table 3.

The TG thermograms of the pure PLLA in Figure 5a showed a single decomposition step of PLLA in the range of 300–450 °C, whereas the pure block copolymer in Figure 5b exhibited two decomposition steps of PLLA and PEG blocks in ranges of 250–350 °C and 350–450 °C, respectively [20]. Increasing the CaCO_3_ ratio of the composites steadily decreased the *5%*–*T_d_* value for the PLLA-based composites. It is interesting that the *5%*–*T_d_* value of the block copolymer shifted dramatically from 282 °C to 302 °C when the 5 %wt CaCO_3_ was loaded, suggesting that the addition of CaCO_3_ significantly slowed down the thermal decomposition of the PLLA blocks in the block copolymer matrices. The *5%*–*T_d_* value of the block copolymer decreased as the CaCO_3_ with higher 5 %wt was loaded. The residue weights at 600 °C of both the pure PLLA and the block copolymer were approximately zero (Table 3), indicating that they were completely thermally decomposed. The value of the residue weight at 600 °C of the composites steadily increased with the CaCO_3_ content because the CaCO_3_ did not decompose below 600 °C [15].

According to Table 3, the PLLA-based composites exhibited single *PLLA*–*T_d,max_* peaks, whereas the block copolymer-based composites showed the *PLLA*–*T_d,max_* and *PEG*–*T_d,max_* peaks. The *PLLA*–*T_d,max_* peak of the PLLA-based composites steadily shifted to a lower temperature as the CaCO_3_ ratio increased. The results of the *5%*–*T_d_* from TG thermograms and the *PLLA*–*T_d,max_* from DTG thermograms indicated that the addition of CaCO_3_ decreased the thermal stability of the PLLA matrices. This may be explained by the CaCO_3_-catalyzed depolymerization of the PLLA by chain scission at the ester bonds [15].

The *PLLA*–*T_d,max_* peak of the block copolymer shifted dramatically from 309 °C to 338 °C when the 5 %wt CaCO_3_ was loaded. The increasing thermal stability of the block copolymer-based composites may have been due to the interactions between the components, as described in the FTIR analysis detailed above. However, the *PLLA*–*T_d,max_* peak of the block copolymer-based composites shifted to a lower temperature when the CaCO_3_ ratio was higher than 5 %wt. At these CaCO_3_ ratios, the effect of CaCO_3_ filler on the thermal stabilization of the block copolymer matrices could have decreased because of the aggregation of CaCO_3_ particles, which reduced the interactions between the components, as indicated by the previously described FTIR and DSC analyses. It should be noted that all the block copolymer-based composites still had the *PLLA*–*T_d,max_* at higher temperature than the pure block copolymer. The *PEG*–*T_d,max_* peak of the block copolymer-based composites shifted slightly to a lower temperature as the CaCO_3_ ratio increased. Overall, the TG and DTG results revealed that the addition of CaCO_3_ improved the thermal stability of the block copolymer matrices but did not improve that of the PLLA matrices.

### 3.4. Crystalline Structures

**Figure 7:**
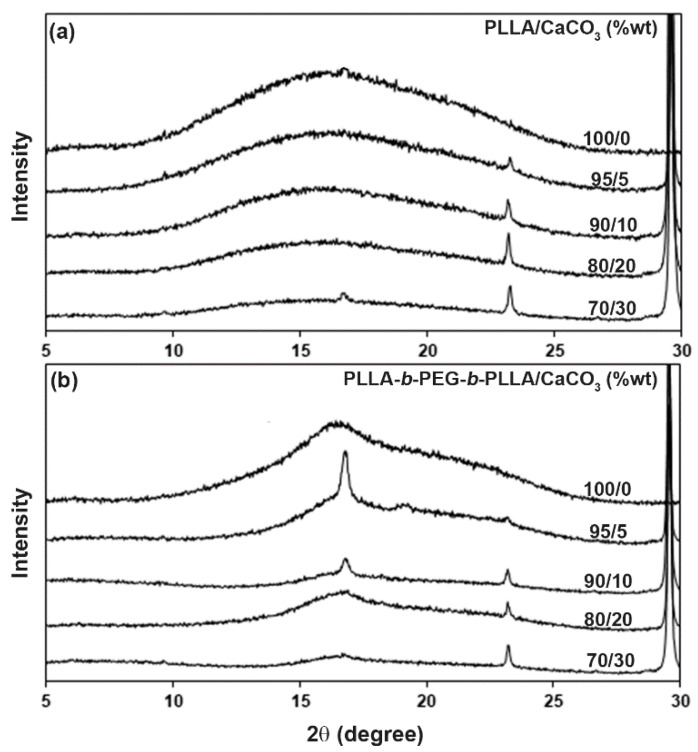
Expanded XRD patterns of (**a**) PLLA/CaCO_3_ and (**b**) PLLA-*b*-PEG-*b*-PLLA/CaCO_3_ composite films with various CaCO_3_ ratios.

XRD patterns were used to investigate the crystalline structures of the composite films, as shown in Figure S5. All the composites exhibited XRD peaks of CaCO_3_ at 2*θ* = 23.2°, 29.5°, 31.6°, 36.1°, 39.5°, 43.3°, 47.6°, and 48.6° [17]. The intensities of these peaks increased with the CaCO_3_ content. Figure 7 shows expanded XRD patterns in the range of 5–30° of the composite films. The pure PLLA film in Figure 7a had no XRD peaks of the PLLA crystallites, suggesting that it was completely amorphous. All the PLLA-based composite films were also completely amorphous except for the 70/30 PLLA/CaCO_3_ composite, which showed a small peak at 2*θ* = 16.5° of the PLLA crystallites [23].

The pure block copolymer in Figure 7b exhibited a broad peak at 2*θ* = 16.5° of the PLLA crystallites. This was due to the PEG middle-blocks enhancing the crystallization of the PLLA end-blocks [9,10]. The peak intensity of PLLA crystallites increased significantly when a 5 %wt CaCO_3_ ratio was added. However, the intensity of this peak decreased when the CaCO_3_ ratio was higher than the 5 %wt. The *XRD* − *X_c_* values of the composites are summarized in Table 4. It was found that the *XRD* − *X_c_* values of the block copolymer-based composites increased as the CaCO_3_ ratio increased, indicating that the added CaCO_3_ enhanced crystallization of PLLA end-blocks. Differences in the *XRD* − *X_c_* and *DSC* − *X_c_* values may be explained by different thermal history and shear force. The strain-induced nucleation influenced the crystallinity of the PLLA [26,27].

### 3.5. Phase Morphology

**Figure 8:**
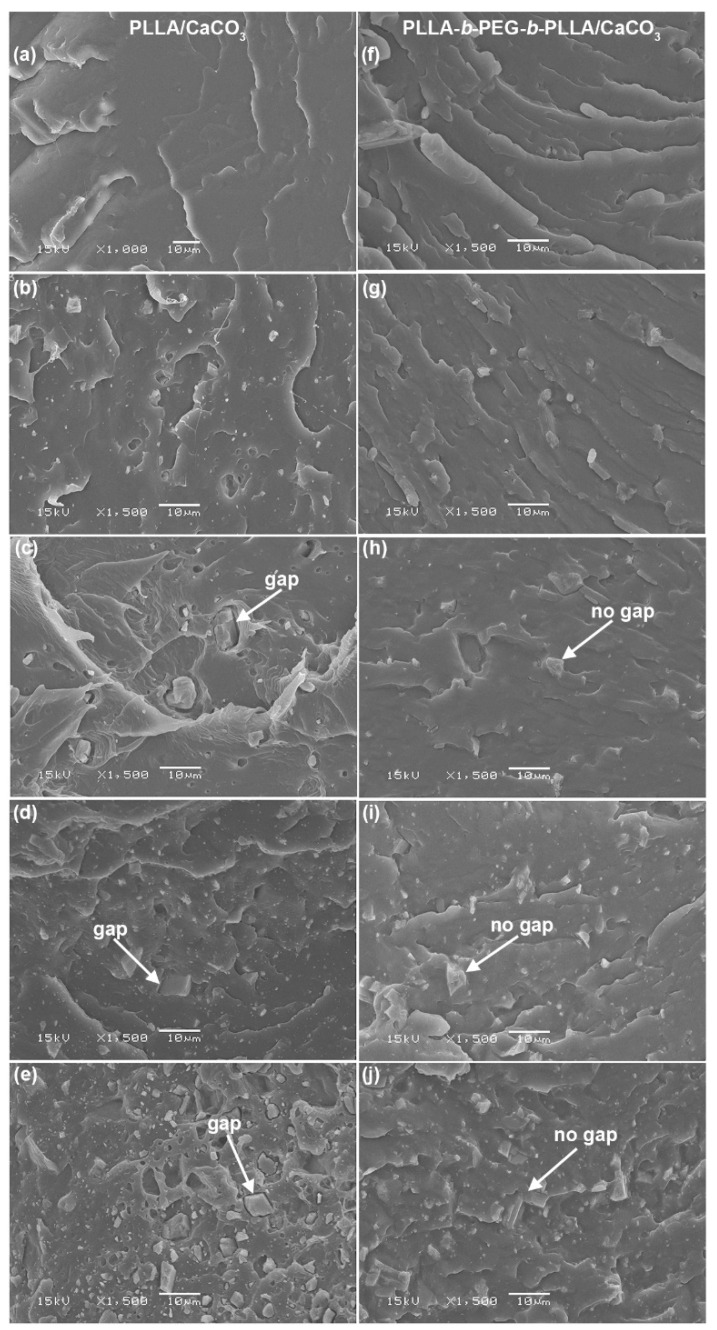
SEM images of cryogenic film fractures with PLLA/CaCO_3_ ratios of (**a**) 100/0, (**b**) 95/5, (**c**) 90/10, (**d**) 80/20, and (**e**) 70/30 %wt, as well as with PLLA-*b*-PEG-*b*-PLLA/CaCO_3_ ratios of (**f**) 100/0, (**g**) 95/5, (**h**) 90/10, (**i**) 80/20, and (**j**) 70/30 %wt (all bar scales = 10 µm).

The phase morphology of the composites was analyzed from SEM images, as illustrated in Figure 8. Phase separation between dispersed CaCO_3_ and film matrices was clearly observed. The amount and aggregation of CaCO_3_ particles increased significantly as the CaCO_3_ ratio increased. The gaps between the CaCO_3_ surfaces and the PLLA matrices were clearly detected, especially for the larger CaCO_3_ particles, as indicated by the white arrows in Figure 8c–e. Some CaCO_3_ particles were detached from the PLLA matrices, which was attributed to the poor phase compatibility between the PLLA matrix and CaCO_3_. Consequently, empty holes were formed, as can be clearly observed in Figure 8e. This is due to the different hydrophilicity resulting in weak interfacial adhesion between the components [16,17]. CaCO_3_ has higher hydrophilicity than PLLA. Interestingly, the block copolymer-based composites had no gaps between the block copolymer matrix and CaCO_3_, indicating that they had good phase compatibility, as shown by the white arrows in Figure 8h–j. This suggests strong interfacial adhesion between components in the block copolymer-based composites compared to the PLLA-based composites. This can be explained by the block copolymer having higher hydrophilicity than the PLLA due to the high hydrophilicity of PEG blocks [20]. The SEM results supported the evidence of interactions between the block copolymer matrix and CaCO_3_ in the above FTIR results.

### 3.6. Tensile Properties

**Figure 9:**
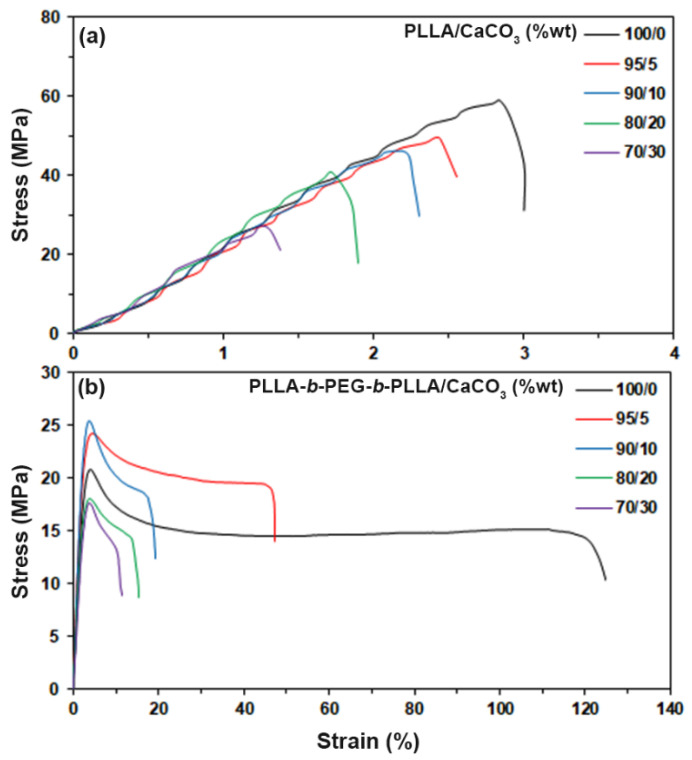
Stress–strain curves of (**a**) PLLA/CaCO_3_ and (**b**) PLLA-*b*-PEG-*b*-PLLA/CaCO_3_ films with various CaCO_3_ ratios.

Tensile testing was used to study the mechanical properties of the composites. Figure 9 shows the stress–strain curves of all the film samples. Averaged tensile properties of the composites are summarized in Table 5. The stress at break, strain at break, and Young’s modulus of the PLLA-based composites decreased as the CaCO_3_ ratio increased, which implies that the addition of CaCO_3_ decreased the mechanical properties of the PLLA. This is because of the weak interfacial adhesion between the components [1], as described in the SEM analysis. The weak interfacial adhesion between the mineral fillers and matrix tends to decrease the tensile stress of the materials due to the fillers acting as the stress concentration points [1,28,29].

The pure block copolymer and its composites exhibited a yield point (Figure 9b), which implies that they were more flexible than the PLLA because the *T_g_* of block copolymer matrix was lower than that of the PLLA matrix (see Table 1). As shown in Table 5, all the block copolymer-based composites showed lower stress at break, a lower Young’s modulus, and higher strain at break than those of the PLLA-based composites. This is due to the PEG middle-block acting as plasticizing sites that enhance the chain mobility of PLLA end-blocks [9,10]. However, it was seen that the stress at yield, stress at break, and Young’s modulus of the pure block copolymer increased and strain at break decreased when the 5 %wt CaCO_3_ was initially added. This indicates that there were effective interactions between the filler and matrix [30,31,32], as supported by the FTIR and SEM results. Therefore, the CaCO_3_ acted as a reinforcing filler for the block copolymer. It was further seen that the stress at yield, stress at break, and Young’s modulus of the block copolymer-based composites decreased as the CaCO_3_ ratio increased beyond 5 %wt. This may be due to the agglomeration of the CaCO_3_ at high ratios resulting in the reduction on the reinforcement of CaCO_3_ in the composites [32,33]. The strain at break of the block copolymer-based composites decreased steadily as the CaCO_3_ ratio increased because of the high rigidity of the CaCO_3_ mineral filler. However, these values were still higher than those of the pure PLLA (3.3%).

## 4. Conclusions

In this study, the PLLA-*b*-PEG-*b*-PLLA block copolymer-based composites were prepared by melt blending with CaCO_3_ and compared to the PLLA-based composites. The results indicated that the molecular interactions between the block copolymer matrix and the CaCO_3_ filler enhanced the PLLA crystallization properties and thermal stability. These properties decreased slightly as the CaCO_3_ contents increased beyond 5 %wt. The block copolymer matrix and CaCO_3_ showed good phase compatibility. The addition of CaCO_3_ at 5 %wt and 10 %wt resulted in the reinforcing effects that increased the tensile stress and Young’s modulus of the block copolymer-based composites. In contrast, CaCO_3_ did not improve these properties of the PLLA-based composites. In conclusion, CaCO_3_ showed great potential for use as a low-cost filler, a nucleating agent, and a reinforcing filler for the flexible PLLA-*b*-PEG-*b*-PLLA bioplastic. These biocomposites have a competitive production cost and can be used as flexible and biodegradable packaging materials.

## Figures and Tables

**Table 1:** Thermal transition properties of the composites from Figure 3.

Sample	*T_g_* (°C) ^a^	*T_cc_* (°C) ^b^	*T_m_* (°C) ^c^	*DSC* − *X_c_* (%) ^d^
PLLA/CaCO_3_ (%wt)				
100/0	58	100	167	9.2
95/5	59	100	168	10.4
90/10	59	100	168	9.8
80/20	60	100	168	11.4
70/30	59	100	168	10.2
PLLA-*b*-PEG-*b*-PLLA/CaCO_3_ (%wt)				
100/0	30	81	159	13.9
95/5	30	75	160	18.4
90/10	30	76	159	18.2
80/20	31	76	160	17.9
70/30	32	78	161	17.1

^a^ Glass transition temperature. ^b^ Cold crystallization temperature. ^c^ Melting temperature. ^d^ Degree of crystallinity from DSC calculated using Equation (1).

**Table 2:** Thermal transition properties of the composites from Figure 4.

Sample	*T_c_* (°C) ^a^	Δ*H_c_* (J/g) ^b^
PLLA/CaCO_3_ (%wt)		
100/0	99	5.5
95/5	96	5.8
90/10	95	5.5
80/20	96	6.3
70/30	96	6.1
PLLA-*b*-PEG-*b*-PLLA/CaCO_3_ (%wt)		
100/0	99	11.7
95/5	112	31.7
90/10	106	28.1
80/20	105	27.5
70/30	105	22.9

^a^ Crystallization temperature. ^b^ Enthalpy of crystallization.

**Table 3:** Thermal decomposition properties of the composites.

Sample	*5%*–*T_d_* (°C) ^a^	Residue Weight at 600 °C (%) ^b^	*PLLA*–*T_d,max_* (°C) ^c^	*PEG*–*T_d,max_* (°C) ^d^
PLLA/CaCO_3_ (%wt)				
100/0	345	0.9	379	-
95/5	335	5.8	369	-
90/10	324	9.8	362	-
80/20	307	21.2	355	-
70/30	305	28.8	351	-
PLLA-*b*-PEG-*b*-PLLA/CaCO_3_ (%wt)				
100/0	282	0.4	309	421
95/5	302	4.7	338	417
90/10	290	10.0	335	419
80/20	285	19.8	324	417
70/30	276	28.7	311	409

^a^ Decomposition temperature at 5% weight loss determined from Figure 5. ^b^ Obtained from Figure 5. ^c^ Decomposition temperature at maximum rate for PLLA obtained from Figure 6. ^d^ Decomposition temperature at maximum rate for PEG obtained from Figure 6.

**Table 4:** Crystallinity contents of the composite films from Figure 7.

Sample	*XRD* − *X_c_* (%) ^a^
PLLA/CaCO_3_ (%wt)	
100/0	-
95/5	-
90/10	-
80/20	-
70/30	1.5
PLLA-*b*-PEG-*b*-PLLA/CaCO_3_ (%wt)	
100/0	10.8
95/5	16.4
90/10	17.5
80/20	17.6
70/30	18.4

^a^ Degree of crystallinity from XRD calculated using Equation (2).

**Table 5:** Averaged tensile properties of the composite films.

Sample	Stress at Yield (MPa)	Stress at Break (MPa)	Strain at Break (%)	Young’s Modulus (MPa)
PLLA/CaCO_3_ (%wt)				
100/0	-	59.4 ± 4.6	3.3 ± 1.2	923 ± 26
95/5	-	52.1 ± 3.1	2.6 ± 0.8	817 ± 31
90/10	-	44.8 ± 3.6	2.5 ± 1.1	605 ± 23
80/20	-	41.7 ± 5.3	1.8 ± 0.6	425 ± 38
70/30	-	27.4 ± 2.6	1.5 ± 0.7	332 ± 16
PLLA-*b*-PEG-*b*-PLLA/CaCO_3_ (%wt)				
100/0	21.8 ± 3.4	18.9 ± 3.2	108.3 ± 8.6	280 ± 27
95/5	25.2 ± 2.6	21.6 ± 2.4	42.1 ± 5.4	396 ± 14
90/10	24.9 ± 4.1	18.5 ± 3.6	20.7 ± 3.1	345 ± 38
80/20	18.5 ± 3.8	13.5 ± 2.1	17.5 ± 2.5	218 ± 23
70/30	17.8 ± 2.7	13.2 ± 1.7	11.6 ± 2.4	219 ± 29

## Data Availability

Not applicable.

## Supplementary Materials

The following supporting information can be downloaded at: https://www.mdpi.com/article/10.3390/polym15020301/s1, Figure S1: GPC curve of a chain-extended PLLA-b-PEG-b-PLLA; Figure S2: 1H-NMR spectrum of a chain-extended PLLA-b-PEG-b-PLLA; Figure S3: FTIR spectrum of CaCO3 powder; Figure S4: Expanded ATR-FTIR spectra of PLLA-b-PEG-b-PLLA/CaCO3 composite films with various CaCO3 ratios; Figure S5: XRD patterns of (a) PLLA/CaCO3 and (b) PLLA-b-PEG-b-PLLA/CaCO3 films with various CaCO3 ratios.
