# Supplementary material for: Phase Morphology, Mechanical, and Thermal Properties of Calcium Carbonate-Reinforced Poly(L-lactide)-b-poly(ethylene glycol)-b-poly(L-lactide) Bioplastics
Source: Polymers (Basel). 2023 Jan 6;15(2):301. doi: 10.3390/polym15020301 (PMC9862968; doi:10.3390/polym15020301)
Supplement: Supplementary file 1 [file polymers-15-00301-s001.zip › polymers-2072623-supplementary.pdf]

Supplementary Materials

Phase Morphology, Mechanical, and Thermal Properties of Calcium Carbonate-Reinforced Poly(L-lactide)-*b*-poly(ethylene glycol)-*b*-poly(L-lactide) Bioplastics

Prasong Srihanam, Wiriya Thongsomboon and Yodthong Baimark *

Biodegradable Polymers Research Unit, Department of Chemistry and Centre of Excellence for Innovation in Chemistry, Faculty of Science, Mahasarakham University, Mahasarakham 44150, Thailand

* Correspondence: yodthong.b@msu.ac.th

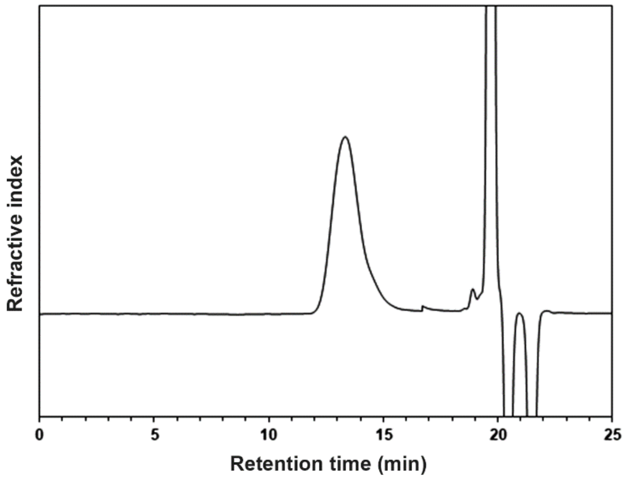

Figure S1. GPC curve of a chain-extended PLLA-*b*-PEG-*b*-PLLA.

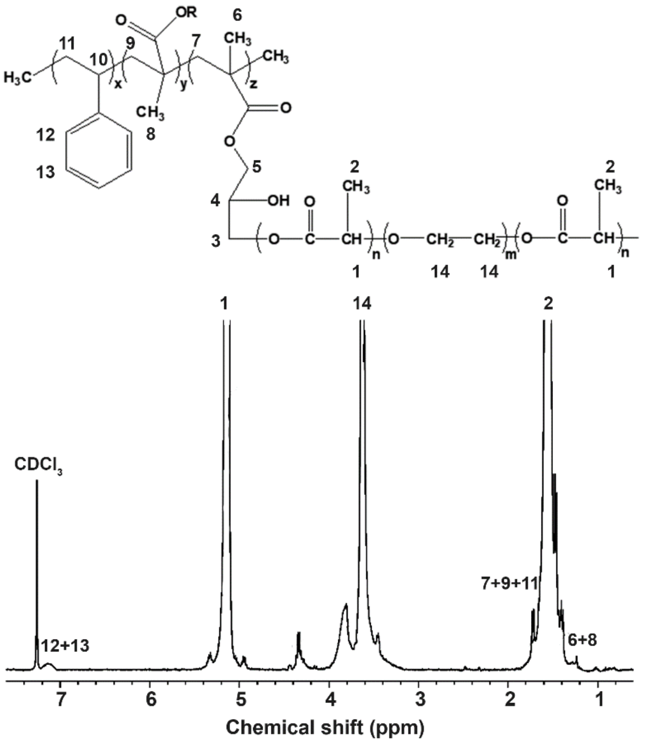

Figure S2. ^{1}H-NMR spectrum of a chain-extended PLLA-*b*-PEG-*b*-PLLA.

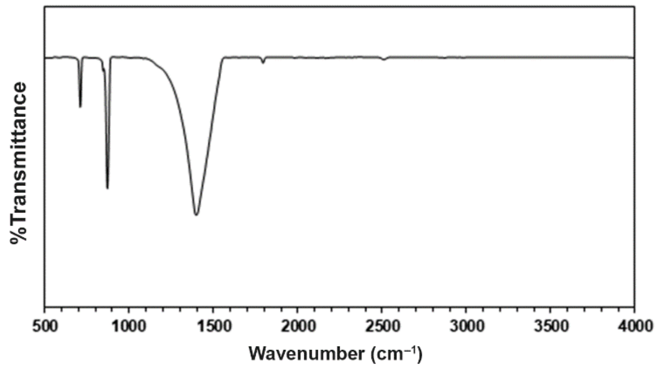

Figure S3. FTIR spectrum of $CaCO_3$ powder.

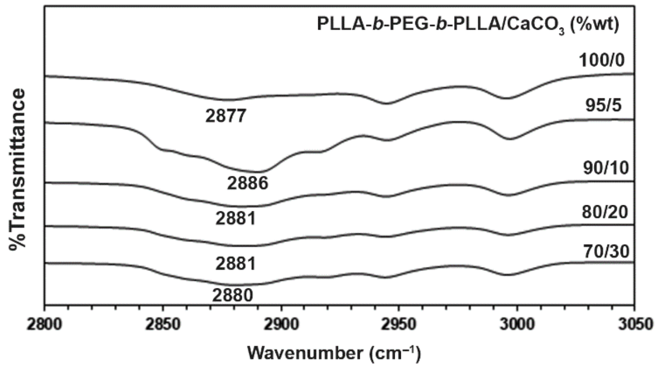

Figure S4. Expanded ATR-FTIR spectra of PLLA-*b*-PEG-*b*-PLLA/$CaCO_3$ composite films with various $CaCO_3$ ratios.

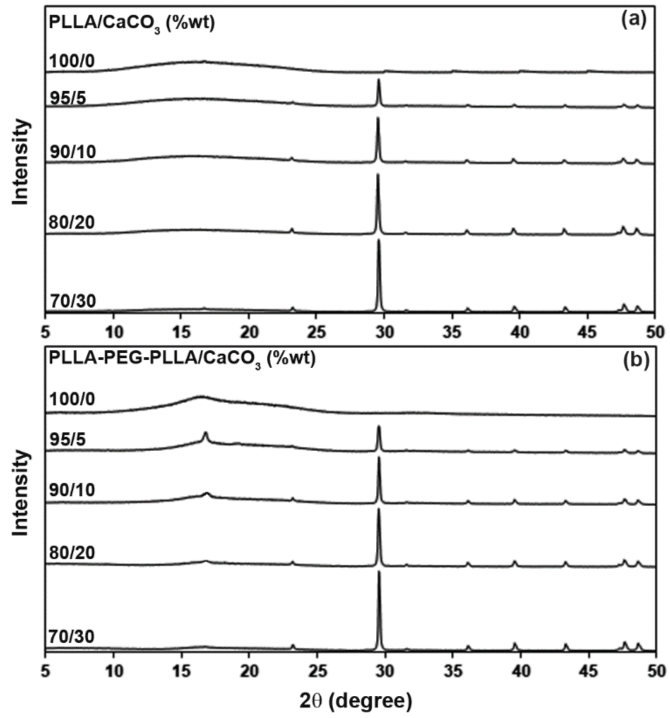

Figure S5. XRD patterns of (a) PLLA/$CaCO_3$ and (b) PLLA-*b*-PEG-*b*-PLLA/$CaCO_3$ films with various $CaCO_3$ ratios.
